# Association of TLR4 Gene rs2149356 Polymorphism with Primary Gouty Arthritis in a Case-Control Study

**DOI:** 10.1371/journal.pone.0064845

**Published:** 2013-05-30

**Authors:** Yu-Feng Qing, Jing-Guo Zhou, Quan-Bo Zhang, Dong-Sheng Wang, Min Li, Qi-Bin Yang, Cui-Ping Huang, Ling Yin, Shu-Yue Pan, Wen-Guang Xie, Meng-Yun Zhang, Meng-Jun Pu, Mei Zeng

**Affiliations:** 1 Department of Rheumatology and Immunology of the Affiliated Hospital, North Sichuan Medical College, China; 2 Department of geriatrics of the Affiliated Hospital, North Sichuan Medical College, China; 3 Department of Clinical Laboratory,The Affiliated Hospital of North Sichuan Medical College, China; 4 Department of genetics, North Sichuan Medical College, China; University of Milan, Italy

## Abstract

**Background:**

The **t**oll-like receptor (TLR)4-interleukin1β (IL1β) signaling pathway is involved in the monosodium urate (MSU)-mediated inflammation. The aim of this present study was to determine whether the TLR4 gene rs2149356 SNP is associated with gouty arthritis (GA) susceptibility and whether rs2149356 SNP impacts the TLR4-IL1β signaling pathway molecules expression.

**Methods and Findings:**

The rs2149356 SNP was detected in 459 GA patients and 669 control subjects (containing 459 healthy and 210 hyperuricemic subjects). Peripheral blood mononuclear cells (PBMCs) TLR4 mRNA and serum IL1β were measured in different genotype carriers, and correlations between TLR4 gene SNP and TLR4 mRNA, IL1β were investigated. The frequencies of the genotype and allele were significantly different between the GA and control groups (*P*<0.01, respectively). The TT genotype was associated with a significantly increased risk of GA (OR = 1.88); this finding was not influenced by making adjustments for the components of possible confounders (adjusted OR = 1.96). TLR4 mRNA and IL1β were significantly increased in the TT genotype from acute GA patients (*P*<0.05, respectively), and lipids were significantly different among three genotypes in the GA patients (*P*<0.05, respectively).

**Conclusions:**

The TLR4 gene rs2149356 SNP might be associated with GA susceptibility, and might participate in regulating immune, inflammation and lipid metabolism. Further studies are required to confirm these findings.

## Introduction

Gout, the most common form of autoinflammatory arthritis among men, is characterized by elevated urate and monosodium urate (MSU) crystal deposition in tissues, which leads to arthritis, the occurrence of soft tissue masses (i.e., tophi), nephrolithiasis, and urate nephropathy [1], [2]. The epidemiological evidence suggests that both the incidence and prevalence of gout are rising [3] **,** and the incidence is 1.14% and 1.4% in the Shandong coastal cities of Eastern China and in the eastern counties, respectively [4], [5]. Although the concrete pathogenesis of gout is still unclear, accumulating evidence indicates that genetic factors, environmental triggers and immune dysregulation may be involved in its development [6]. Attacks of gout are triggered by the deposition of MSU crystals in the joints and MSU crystals are widely recognized as endogenuos “danger signal” by components of the innate immune system [7], [8].

The studies have demonstrated that genetic polymorphisms of SLC2A9, URAT1 are key regulators of urate homeostasis, the inheritance of one predisposing variant of SLC2A9 or URAT1 increases the risk of an individual’s developing gout [6], [9], [10]. However, an epidemiological study found that only about 10% of hyperuricemia patients develop gout [11]. Why are certain hyperuricemic individuals predisposed to gout? One possible explanation is that other genes that are unrelated to urate metabolism also contribute to an individual’s susceptibility to this disease. Toll-like receptors (TLRs) are transmembrane proteins that are expressed by cells of the innate immune system; they recognize pathogen-associated molecular patterns and play important roles in immune and inflammatory responses to destroy the invaders. Among TLR family members, TLR4 has been the most thoroughly investigated. In addition to its involvement in the recognition of lipopolysaccharide, TLR4 also interacts with endogenous ligands, including MSU [7], [8], [12]–[14] and heatshock proteins (HSPs) [15]. It has been demonstrated that some auto-inflammatory diseases including rheumatoid arthritis, crohn's disease and ulcerative colitis, bronchial asthma and atopic dermatitis, are associated with single nucleotide polymorphisms (SNPs) in the TLR4 gene [16]–[19]. Recently, some studies found that individuals with minor allele of TLR4 gene rs2149356 were strongly associated with the occurrence of sepsis in preterm infants, prostate cancer and normal tension glaucoma [20]–[22]. However, no study has comprehensively investigated the association between rs2149356 of TLR4 gene polymorphism and GA susceptibility or whether rs2149356 SNP might impact the expression of TLR4-IL11β signaling pathway molecules in the peripheral blood of GA patients.

We conducted this study to investigate TLR4 gene rs2149356 polymorphism in GA patients and control subjects (the latter included both healthy and hyperuricemic individuals) and to explore whether rs2149356 polymorphism contribute to GA susceptibility in the Chinese Han population. Additionally, we intended to determine whether the rs2149356 SNP impacts the expression of TLR4-IL11β signaling pathway molecules in peripheral blood from patients with gout.

## Materials and Methods

### Populations

Four hundred and fifty-nine consecutive male patients with primary gouty arthritis (GA) who visited the Department of Rheumatology, the Affiliated Hospital of North Sichuan Medical College between June 2008 and February 2013 were included in the study. The patients were confirmed suffering from GA according to the American College of Rheumatology (ACR) classification criteria (1977) [23]; in addition, they had no history of cancer, hematopathy, nephropathy, infection or other autoimmune diseases. GA patients were divided into an acute gouty arthritis (AGA) group and a non-acute gouty arthritis (NAGA) group based on whether patients are presenting onset of symptoms or not. The GA patients were not receiving any systemic anti-inflammatory treatment, or drugs to control the production and elimination of uric acid before the blood samples were obtained.

Clinical data were carefully recorded, and a medical history was obtained from all of the GA patients, including details of previous episodes of GA, any associated systemic diseases, and the previous use of anti-inflammatory medications or agents used to control the production and elimination of uric acid. A total of 669 age-matched men without gout (NGA, including 459 healthy and 210 hyperuricemic subjects) who underwent regular physical examination at the Affiliated Hospital of North Sichuan Medical College between June 2008 and February 2013 were included as control subjects in this study. Hyperuricemia was defined as a serum uric acid of ≥417µmol/L. They were no history of any systemic inflammatory disease (Table 1). All the participants are from the Chinese Han population. Blood samples were obtained from all the participants, collected into sterile, anticoagulant-coated tubes and immediately transported to the laboratory for genetic analysis and detection of gene expression and cytokine production.

**Table 1:** Characteristics of gout cases and control study samples (mean ± SD).

	GA group(n = 459)	HUA group (n = 210)	HC group (n = 459)	*P* value
Age(years)	48.18±12.16	49.09±12.23	47.35±11.28	>0.05
Gender(Male/Female)	459/0	210/0	459/0	>0.05
Disease duration (years)	11.53±6.28	–	–	–
Alcohol Consumption, n (%)^f^	365 (79.5)	160 (76.2)		<0.05
Tophi, n (%)	54 (11.8)	–	–	–
Hypertension, n (%)^t^	163 (35.5)a,B	73 (34.8)^a^	83 (18.1)	<0.05
Hypercholesterolemia, n (%)^c^	325 (70.8%)a,B	145 (69.5%)^a^	185(40.3%)	<0.05
Purine-rich foods intake, n (%)^p^	328 (71.5%)a,B	153 (72.9%)^a^	232 (50.5%)	<0.05
BMI(kg/m^2^)*	26.59±3.52a,B	25.68±4.19^a^	23.06±4.45	<0.01
sUA (µmol/L)*	509±163.6a,B	508±68.8^a^	307±58.3	<0.01
GLU(mmol/L)*	6.25±1.81a,B	5.98±0.80^a^	5.23±0.47	<0.01
WBC(×10^9^/L)*	6.65±2.51a,b	4.58±1.18^A^	4.70±1.53	<0.01
GR(×10^9^/L)*	4.75±2.26a,b	3.56±0.68^A^	3.50±0.89	<0.01
LY(×10^9^/L)*	1.79±0.73A,B	1.98±0.72^A^	2.14±0.66	>0.05
Mo(×10^9^/L)*	0.69±0.26a,B	0.50±0.16^A^	0.49±0.18	<0.01
TG(mmol/L)*	2.33±1.62a,B	2.28±0.98^a^	1.16±0.66	<0.01
GLOB (g/L)*	29.68±5.20a,b	26.49±3.88^A^	26.66±4.32	<0.01
TC(mmol/L)	4.88±0.96A,B	4.88±0.86^A^	4.45±0.43	>0.05
HDL(mmol/L)*	1.17±0.43a,b	1.30±0.38^A^	1.35±0.31	<0.05
LDL(mmol/L)*	2.63±0.87A,b	3.38±0.66^a^	2.65±0.55	<0.05
VLDL(mmol/L)*	1.13±0.69a,B	1.11±0.56^a^	0.53±0.22	<0.01
apoA1(mmol/L)*	1.30±0.35A,B	1.28±0.26^A^	1.24±0.20	>0.05
apoB100(mmol/L)*	0.92±0.26a,B	0.89±0.13^a^	0.78±0.14	<0.05
ESR(mm/h)	33.66±23.59	−	−	−
CRP(mg/L)	25.36±36.21	−	−	−

* one-way ANOVA, *P*<0.05. LSD method:

a *P*<0.05,

A *P*>0.05 (in comparison with HC group);

b *P*<0.05,

B *P*>0.05 (in comparison with HUA group). Statistical significance was set at *P*≤0.05.

t Defined as blood pressure ≥140/90 mmHg or ongoing antihypertensive therapy.

c Defined as TG ≥155 mg/dL or intake of lipid lowering medication.

f,t,c,p χ^2^ test:

a *P*<0.05 (in comparison with HC group);

B *P*>0.05 (in comparison with HUA group). GA: gouty arthritis; HUA: hyperuricemia without gout; HC: healthy control subjects; BMI: Body Mass Index, sUA: serum uric acid; GLU: serum glucose; WBC: white blood cell counts; GR: neutrophile granulocytecounts; LY: lymphocyte counts; Mo: monocyte counts; TG: triglycerides; TC: Total Cholesterol; HDL: high density lipoprotein; LDL: low density lipoprotein; VLDL: very low density lipoprotein; apoA1: apolipoprotein A1; apoB100: apolipoprotein B100; ESR: erythrocyte sedimentation rate; CRP: C reactive protein.

The Ethics Committee of the Affiliated Hospital of the North Sichuan Medical College approved the study protocol and all the participants gave their written informed consent to participation in the study at the time of inclusion and again at the time of follow-up investigations. The study was conducted in accordance with the principles of the current version of the Declaration of Helsinki. The design, analysis and interpretation of the present study are based on previous reports [24].

### Laboratory Examination of Regulatory Parameters

Plasma total cholesterol (TC), triglycerides (TG), high density lipoprotein cholesterol (HDL), low density lipoprotein cholesterol (LDL), very low density lipoprotein (VLDL), apolipoprotein A1(apoA1) and apolipoprotein B100 (apoB100) and serum uric acid (sUA) were measured using a 7170S autoanalyzer (Hitachi Co, Tokyo, Japan) by an expert who blinded to the study. The erythrocyte sedimentation rates (ESR), the blood cells count, C-reactive protein (CRP) level and serum glucose (GLU) level were routine laboratory tests that were performed. All of the measurements were carried out by the Department of Clinical Laboratory, the Affiliated Hospital of North Sichuan Medical College.

### DNA Isolation and Genetic Analyses

Genomic DNA was isolated from whole blood samples of 459 GA cases and 669 control subjects, including 459 healthy subjects and 210 hyperuricemic subjects, using the Pure Gene DNA Blood Kit (Gentra, Minneapolis, MN, USA). DNA samples were genotyped using 5′ exonuclease TaqMan® technology (Applied Biosystems, Foster City, CA, USA).

All of the genotyping assays were designed by Applied Biosystems (Foster City, CA, USA).

The genotypes were assigned by an investigator who was blinded to the patients’ clinical status.

The genotyping reaction utilizes two dual-labeled TaqMan probes that specifically target the alternate alleles. The two probes are labeled with a fluorescent reporter dye (VIC or FAM) and a non-fluorescing quencher/minor groove binder (MGB). When a probe specifically binds to the SNP site, the 5′ nuclease activity of the Taq polymerase during the PCR allows for the cleaving and subsequent fluorescence of the reporter dye. At the conclusion of the PCR, the samples were genotyped by analysis of the fluorescence of the two dyes. Each 5.0 µl PCR contained the following: TaqMan® Universal PCR Master Mix, No AmpErase® UNG (2X), Assays-on-Demand™ (20X) or Assays-by-Design™ (40X) SNP Genotyping Assay Mix, and 1 ng of genomic DNA. Assays were conducted in a 96-well format on the ABI PRISM® 7900HT Sequence Detection System (Applied Biosystems). Reaction conditions were the following: initial denaturation at 95°C for 10 min, followed by 40 cycles each of denaturation (92°C for 15 s) and annealing/extension (60°C for 60 s). Case and control DNA was genotyped together on the same plates with duplicates samples (15%) to assess intraplate variation and interpolate genotype quality. No genotyping discrepancies were detected. The methods are according to the previous study [25].

### RNA Extraction and Real-Time Quantitative PCR (RT-qPCR) amplification

Peripheral blood mononuclear cells (PBMCs) were isolated using Ficoll-Hypaque density gradient centrifugation from the blood samples of 90 acute GA patients (30 GG, 35 GT and 25 TT genotypes carriers) and 120 non-acute GA patients (35 GG, 55 GT and 30 TT genotypes carriers), 108 healthy subjects (33 GG, 50 GT and 25 TT genotypes carriers) and 96 hyperuricemic subjects (30 GG, 40 GT and 26 TT genotypes carriers). Total RNA was extracted from PBMCs using Trizol reagent (Invitrogen, USA) and reverse-transcribed into cDNA using reagents that included random hexamers, superscript II, and dNTP (Invitrogen, USA). The converted cDNA was cryopreserved at –80°C until RT-qPCR was performed.

RT-qPCR was carried out with a final volume of 20 µl in the ABI Prism 7900HT Sequence Detection System (Applied Biosystems, USA). The reaction contained Power SYBR Green PCR Master Mix (Applied Biosystems, USA) (9 µl), 10pmol/L each of forward and reverse primers (0.5 µl each), synthesized cDNA sample (1.3 µl) and ddH2O (8.7 µl). The thermal cycling conditions comprised an initial denaturing step at 95°C for 10 minutes, 40 cycles of renaturation at 95°C for 15 seconds and elongation at 60°C for 1 minute. The PCR reaction for each gene was duplicated for each sample and the mean value was used for further analysis. Additionally, the RT-qPCR reaction was run according to a modification of the Cawthon method [26], [27]. The sequences of the primers used for PCR are given in Table 2.

**Table 2:** Sequences of primer used in the RT-qPCR assays.

Gene	Forward primer	Reverse primer
TLR4	TCCCTGAACCCTATGAAC	CTAAACCAGCCAGACCTT
β-actin	GAGCTACGAGCTGCCTGACG	GTAGTTTCGTGGATGCCACAG

We used relative quantification to evaluate the expression of selected genes [28]; the housekeeping gene β-actin was used as an internal control to normalize the mRNA expression of each target gene.

### ELISA of IL1β

After centrifugation, serum samples were stored at −80°C. IL1β level in the serum obtained from 108 acute GA cases (including 35 GG, 49 GT and 24 TT genotypes carriers), 108 healthy subjects (including 33 GG, 50 GT and 25 TT genotypes carriers) and 96 hyperuricemic subjects (including 30 GG, 40 GT and 26 TT genotypes carriers) was determined using enzyme immunoabsorbent assay (ELISA) kits (R&D System). The detection limits of the IL1β assay was 5–200 pg/ml. The optical density was determined using a microplate reader (Model 3550, Bio-Rad). A standard curve of cytokine IL1β was established using a known concentration of IL1β by plotting the optical density *vs* the log of the concentration.

### Statistical Analysis

Statistical analysis was performed using SPSS (Statistical Package for the Social Sciences) 16.00. The genotype distribution was analyzed for deviations from the Hardy-Weinberg equilibrium (HWE) using χ^2^ analyses. Differences in the allele and genotype frequencies between the cases and the control subjects were assessed using the χ^2^ test with a 3×2 or 2×2 contingency. Associations between genotypes and GA were estimated by computing the odds ratios (OR) and their 95% confidence intervals (CI) from multivariate logistic regression analysis with the adjustment for alcohol consumption, dietary factors, hypercholesterolemia, hypertension, hyperuricemia and BMI, which are possible confounders. One-way ANOVA (analysis of variance) in conjunction with the LSD method for multiple comparisons was performed. All the statistical tests were two-sided at a significance level of 0.05. The statistical power was calculated using PS software (http://biostat.mc.vanderbilt.edu/twiki/bin/view/Main/PowerSampleSize).

## Results

### Clinical and Laboratory Characteristics of the Subjects Studied

The clinical and laboratory data of the subjects are summarized in Table 1. In our cohorts, gout cases were matched by age and gender to control individuals. Specifically, 54 (11.8%) of 459 GA patients had tophi. Significant differences were observed among the GA, HUA (hyperuricemic subjects) and HC (healthy control subjects) groups, in body mass index (BMI), white blood cell counts (WBC), neutrophile granulocyte counts (GR), monocyte counts (Mo), globulin (GLOB), sUA, GLU, TG, LDL, VLDL, HDL and apoB100 (*P*<0.05, respectively). WBC, GR, Mo and GLOB were significantly increased in the GA patients compared to HUA and HC subjects (*P*<0.05, respectively; Table 1); sUA, BMI, GLU, TG, VLDL, and apoB100 were much higher in GA patients and HUA subjects than those in HC subjects (*P*<0.05, respectively; Table 1), whereas in HC and HUA subjects, the HDL level was elevated (*P*<0.05, respectively; Table 1). The LDL level was significantly increased in the HUA group compared to the GA and HC group (*P*<0.05, respectively; Table 1).

### Association of the TLR4 Gene rs2149356 SNP with Risk of Gout

The genotyping success rate was 100% in our study cohort. The Genotypes distributions and allele frequencies in the gout case-control cohort are shown in Table 3. There were no significant deviations from HWE both in GA and control groups (*P*>0.05, respectively; Table 3). The minor allele frequency (MAF) of the SNP was 20.7%, which is consistent with that reported in the HapMap database (http://www.hapmap.org).

**Table 3:** Distributions of genotypes of the TLR4 gene rs2149356 and their associations with risk of GA.

Variables	GA (n = 459)	NGA (n = 669)	χ^2^	*P*	OR (95%CI)	OR (95%CI)^A^
GG^ Reference^	123 (26.8%)	245 (36.62%)	–	–	1.00	1.00
GT	220 (47.9%)	306 (45.74%)	6.46	0.011	1.43 (1.09–1.89)	1.09 (0.88–1.82)
TT	116 (25.3%)	118 (17.64%)	15.58	7.9×10^−5^	1.88 (1.33–2.65)	1.96 (1.40–2.74)
HWE^P^	0.378	0.187				
G allele ^Reference^	466 (50.8%)	796 (59.5%)	–	–	1.00	
T allele	452 (49.2%)	542 (40.5%)	16.83	4.1×10^−5^	1.42 (1.20–1.69)	
	GA (n = 459)	HC (n = 459)				
GG^ Reference^	123 (26.8%)	173 (37.7%)	–	–	1.00	1.00
GT	220 (47.9%)	208 (45.3%)	6.81	0.009	1.49 (1.10–2.01)	1.12 (0.99–1.92)
TT	116 (25.3%)	78 (17.0%)	15.60	7.81×10^−5^	2.00 (1.37–2.92)	2.09 (1.45–3.02)
HWE^P^	0.378	0.255				
G allele^ Reference^	466 (50.8%)	554	–	–	1.00	
T allele	452 (49.2%)	364	17.08	3.58×10^−5^	1.48 (1.23–1.78)	
	GA (n = 459)	HUA (n = 210)				
GG^ Reference^	123 (26.8%)	72 (34.3%)	–	–	1.00	1.00
GT	220 (47.9%)	98 (46.7%)	2.03	0.15	1.31 (0.90–1.91)	1.03 (0.79–1.80)
TT	116 (25.3%)	40 (19.0%)	5.08	0.024	1.64 (1.02–2.62)	1.70 (1.07–2.70)
HWE^P^	0.378	0.519				
G allele^ Reference^	466 (50.8%)	242 (57.6%)	–	–	1.00	
T allele	452 (49.2%)	178 (42.4%)	3.86	0.028	1.26 (1.00–1.60)	

HWE^P^: P value of Hardy-Weinberg equilibrium (HWE);

A Adjusted for alcohol consumption, dietary factors, hypercholesterolemia, hyperuricemia, hypertension and BMI in logistic regression model. GA: gouty arthritis, NGA: subjects without gout (including 459 healthy subjects and 210 hyperuricemic subjects), HUA: hyperuricemia without gout, HC: healthy subjects, OR: odds ratio; CI: confidence interval.

Significant differences were observed between the GA and control groups with respect to genotype and allele frequencies (GA *vs* NGA: χ^2^ = 15.98, 16.83, *P* = 0.0003, 4.08×10^−5^; GA *vs* HC: χ^2^ = 16.23, 17.08, *P* = 0.0003, 3.58×10^−5^; GA *vs* HUA: χ^2^ = 5.22, 3.86, *P* = 0.02, 0.03). As shown in Table 3, the frequencies of the TT genotype and the T allele were significantly increased in the GA group compared with the control groups (*P*<0.05, respectively; Table 3). With the current sample size (459 GA and 669 NGA as controls), an OR of 1.42 with an exposure frequency of 40.5% was detected with 82% power at a significance level of 0.05. Multivariate logistic regression analysis revealed that significantly increased risk of GA was associated with the TT genotype (adjusted OR = 1.96, 95% CI = 1.40–2.74; Table 3), compared with the GG genotype.

There were no differences in the genotype or allele frequencies between 459 healthy and 210 hyperuricemic subjects (χ^2^ = 0.86, 1.66, *P* = 0.65, 0.20; respectively).

### Association of the TLR4 Gene rs2149356 SNP with PBMCs TLR4 mRNA Level and Serum IL1β in Gout Patients

**Figure 1:**
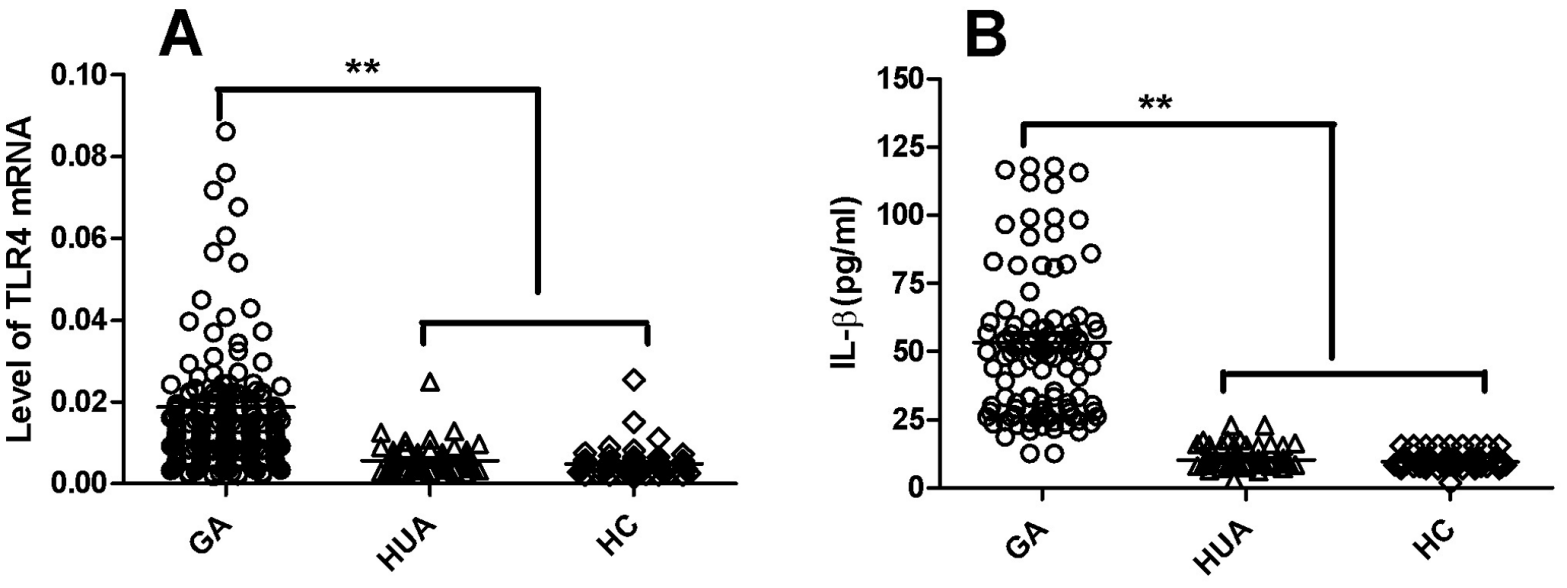
Levels of PBMCs TLR4 mRNA and serum IL1β in the GA, HC and HUA groups. **A)** Expression of PBMCs TLR4 mRNA in 210 GA patients, 108 healthy and 96 hyperuricemic subjects was detected using RT-qPCR. TLR4 mRNA level was significantly increased in the GA group compared with both the HC and HUA groups (*P*<0.01; respectively), whereas no significant difference was observed between the HC and HUA groups (*P*>0.05). **B)** Serum IL1β was measured using ELISA in 108 AGA patients, 108 healthy and 96 hyperuricemic subjects. Expression of serum IL1β was much higher in the AGA patients than in either the healthy or the hyperuricemic subjects (*P*<0.01; respectively), while there was no difference between the HC and HUA groups (*P*>0.05). GA represents gouty arthritis; HC represents healthy subjects; HUA represents hyperuricemia without gout. The ANOVA, LSD method was used. **P<0.01. The statistical significance was set at *P*≤0.05.

**Figure 2:**
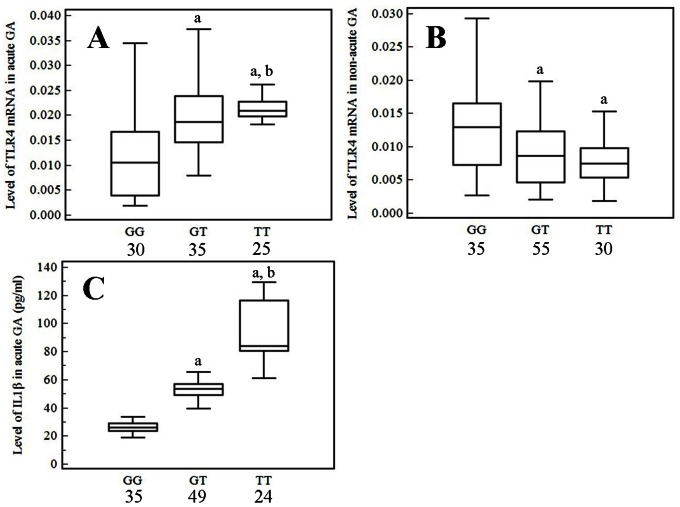
Association of the TLR4 gene rs2149356 SNP with PBMCs TLR4 mRNA and serum IL1β levels in gout patients. The levels of TLR4 mRNA in PBMCs and of IL1β in serum were measured in different genotype carriers from GA patients. **A)** In acute GA patients, the TLR4 mRNA expression level in 25 TT homozygotes and 35 heterozygotes carriers was significantly increased compared with 30 GG homozygotes carriers (*P*<0.01; respectively); and TLR4 mRNA level was higher in TT homozygotes carriers than that in GT homozygotes carriers (*P*<0.01). **B)** In non-acute GA patients, the TLR4 mRNA level was significantly reduced in 30 TT homozygotes or 55 GT heterozygotes carriers compared with 35 GG homozygotes carriers (*P*<0.02, respectively), no difference was observed between GT and TT genotypes (*P*>0.05). **C)** In acute GA patients, the serum IL1β level was much higher in 24 TT and 49 GT carriers than that in 35 GG carriers (*P*<0.01, respectively), and it was higher in TT carriers than that in GT carriers (*P*<0.01). The data are shown as box plots. Each box represents the upper and lower interquartile range (IQR). The wshiskers represent 1.5 times the upper and lower IQRs. The ANOVA, LSD method was performed. ^a^ *P*<0.01 in comparison with patients with the GG genotype; ^b^ *P*<0.01 in comparison with patients with the GT genotype. The statistical significance was set at *P*≤0.05.

The levels of TLR4 mRNA in PBMCs and of IL1β in serum were significantly increased in the GA group compared with the HC and HUA groups (*P*<0.01, respectively; Figure 1), whereas the differences between the HC and HUA groups were not statistically significant (*P*>0.05, respectively; Figure 1). The levels of TLR4 mRNA and serum IL1β in acute GA patients differed significantly among the three genotypes (*F* = 4.633, 6.716; *P = *0.013, 0.008; respectively), TLR4 mRNA level in PBMCs from non-acute GA patients also differed significantly among the three genotypes (*F* = 8.668; *P = *0.006). As shown in Figure 2, we found that, among acute GA patients, PBMCs TLR4 mRNA expression and serum IL1β in TT homozygotes and heterozygotes carriers were significantly higher than those in GG homozygotes carriers (*P*<0.01, respectively; Figure 2); they were also higher in TT homozygotes carriers than those in GT homozygotes carriers (*P*<0.01, Figure 2). In non-acute GA patients, PBMCs TLR4 mRNA was significantly reduced in TT homozygotes and GT heterozygotes carriers compared with GG homozygotes carriers (*P*<0.02, respectively; Figure 2); no difference was observed between GT and TT genotypes carriers (*P*>0.05, Figure 2). There were no differences in PBMCs TLR4 mRNA and serum IL1β in healthy or hyperuricemic subjects of the three different genotypes (TLR4 mRNA in HC and HUA: *F* = 1.492, 0.245, *P* = 0.230, 0.783; IL1β in HC and HUA: *F* = 0.688, 0.882, *P* = 0.505, 0.417).

### Association of the TLR4 Gene rs2149356 SNP with Serum Lipid and GLOB Levels in GA Patients

**Figure 3:**
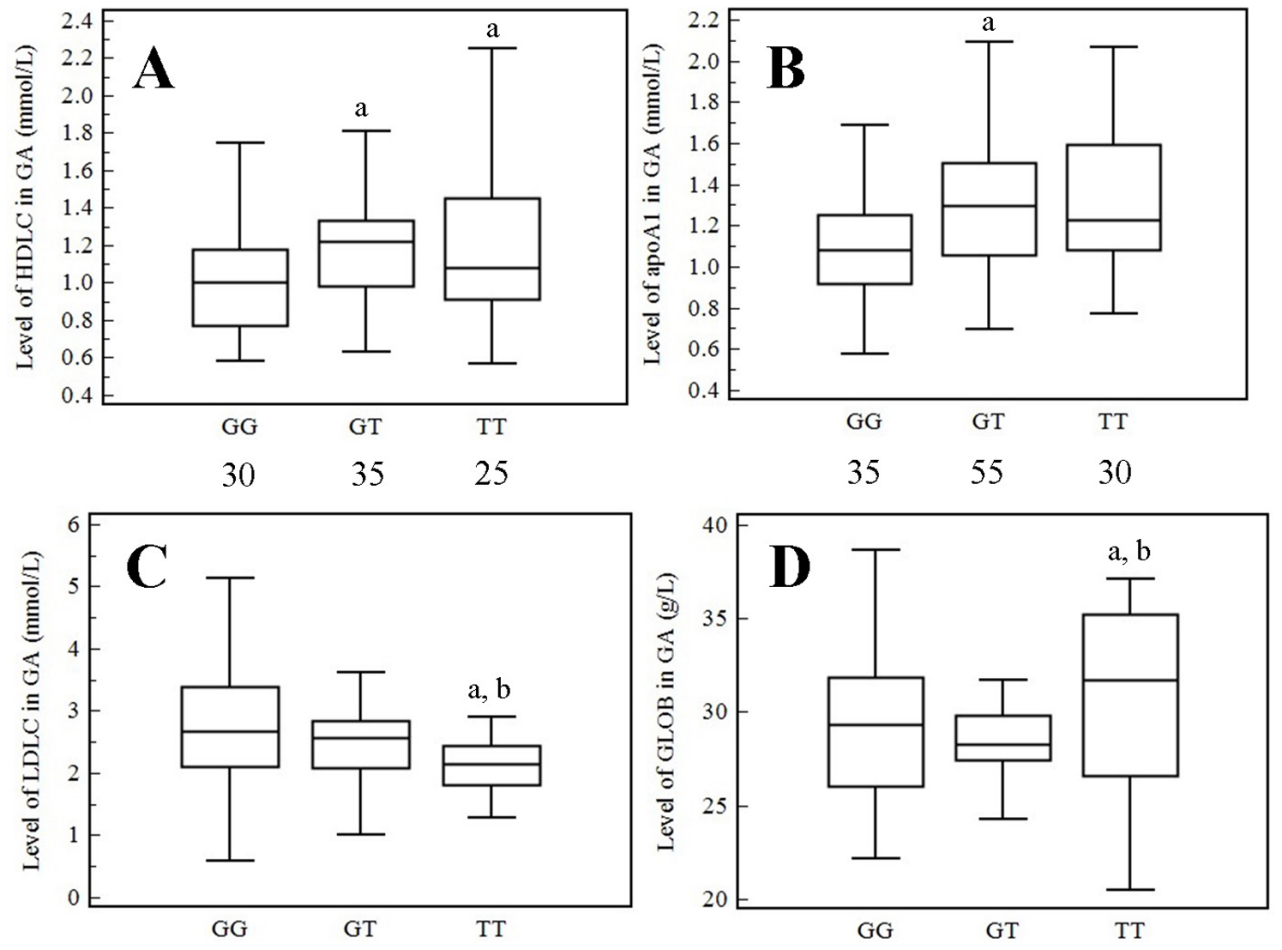
Association of the TLR4 gene rs2149356 SNP with serum lipid and GLOB levels in GA patients. Significant differences were observed among the three genotypes in GA patients with respect to HDL, apoA1, LDL and GLOB levels (*F* = 4.28, 5.13, 3.68, 4.28; *P = *0.016, 0.007, 0.028, 0.016; respectively). **A)** The 35 heterozygotes and 25 TT genotype carriers had higher HDL level than the 30 GG genotype carriers (*P*<0.05, respectively); there was no difference between patients with the GT and TT genotypes (*P*>0.05). **B)** ApoA1 was much higher in 55 GT heterozygotes carriers than that in 35 GG homozygotes carriers (*P*<0.05), and no significant differences were observed between patients with the TT and GG, the TT and GT genotypes (*P*>0.05; respectively). **C)** LDL level was significantly reduced in 25 TT homozygotes carriers compared with 30 GG homozygotes carriers (*P*<0.05), and there were no differences in LDL level between patients carrying 30 GG and 35 GT, 25 TT and 35 GT carriers (*P*>0.05; respectively). **D)** GLOB level was much higher in 30 TT carriers than that in 35 GG or 55 GT carriers (*P*<0.05, respectively), and no significant difference was observed between the GG and GT genotypes (*P*>0.05). The data are shown as box plots. Each box represents the upper and lower interquartile range (IQR). The whiskers represent 1.5 times the upper and lower IQRs. The ANOVA, LSD method was performed. ^a^ *P*<0.05 in comparison with patients with the GG genotype; ^b^ *P*<0.05 in comparison with patients with the GT genotype. The statistical significance was set at *P*≤0.05.

Interestingly, in GA patients, significant differences were observed among the three genotypes with respect to HDL, apoA1, LDL and GLOB (*F* = 4.28, 5.13, 3.68, 4.28; *P = *0.016, 0.007, 0.028, 0.016; respectively). The patients with GT and TT genotypes had higher HDL level than the patients with GG genotype (*P* = 0.007, 0.035, respectively; Figure 3A), while there was no difference in HDL level between patients with GT and TT genotypes (*P* = 0.730; Figure 3A). ApoA1 was much higher in patients with GT genotype than that in GG genotype (*P* = 0.002; Figure 3B), and significant differences were not observed between patients with TT and GG, TT and GT genotypes (*P* = 0.094, 0.238, respectively; Figure 3B). The patients carrying GG genotype had higher LDL level than TT genotype (*P* = 0.007; Figure 3C), and there were no differences in LDL level between patients carrying GG and GT, TT and GT genotypes (*P* = 0.214, 0.110, respectively; Figure 3C). GLOB was much higher in patients with TT genotype than that in GG or GT genotypes (*P* = 0.002, 0.001, respectively; Figure 3D), and no significant difference was observed between GG and GT genotypes (*P* = 0.910; Figure 3D). No significant difference was found among the three genotypes with respect to sUA concentration (*F = *1.259, *P* = 0.287). In healthy and hyperuricemic subjects, there were no differences among the three genotypes with respect to any of the above parameters (*P*>0.05, respectively).

### Comparison of Clinical and Laboratory Data between GA Patients with and without Tophi

No significant differences were observed between GA patients with and without tophi with respect to the genotype or allele frequency (χ^2^ = 1.57, 0.91, *P*>0.05; respectively). Age, disease duration and sUA concentration were much higher in patients with tophi than those without tophi (*P*<0.05, respectively; Table 4). No obvious difference was found between patients with tophi and those without tophi with respect to other clinical and laboratory data (*P*>0.05, respectively).

**Table 4:** Comparison of clinical and laboratory data between GA patients with and without tophi.

	Patients with tophi group(n = 54)	Patients without tophi group (n = 405)	*P* value
Age (years)	56.08±12.87	50.25±11.06	<0.05
Disease duration (years)	11.90±8.04	6.20±7.41	<0.05
sUA (µmol/L)	565.05±134.23	456.34±104.76	<0.05

## Discussion

**Figure 4:**
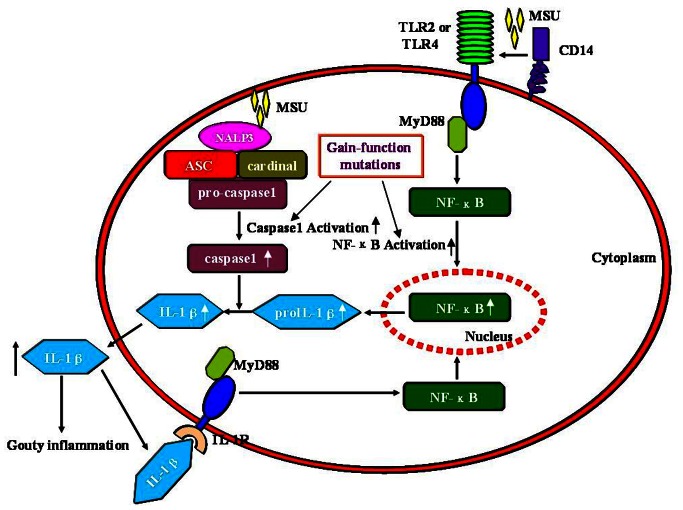
Innate immunity and gout susceptibility genes. Innate immunity components associated with TLR2, TLR4, CD14 and NALP3 inflammasome are involved in MSU-mediated inflammation through activation of TLRs-MyD88 dependent NFκB signaling, the NALP3 inflammasome and IL1β-MyD88 dependent IL1R signaling. Gain-of-function mutations in TLR2, TLR4 or CD14 will enhance MSU-mediated TLRs-NFκB activation, increasing the synthesis of proIL1β; the gain-of-function mutations in the NALP3 inflammasome, which include NALP3 and CARD8, will enhance MSU-mediated NALP3 inflammasome activation and increase IL1β processing. Elevated IL1β is involved in gouty inflammation.

The TLRs-NFκB, NLRP3 inflammasome and IL1β/IL1R pathways play key roles in the initiation and amplification of IL1β-mediated inflammation in acute gout attack [12] (Figure 4). The potential pivotal role of the components of innate immunity, especially TLR2/TLR4 and the NLRP3 inflammasome, in this disease, seems attractive as potential target candidates for the treatment of gout.

Recent findings underscore the importance of the TLR4 gene rs2149356 SNP and some inflammatory diseases susceptibility [16]–[19]. However, the association between TLR4 gene rs2149356 SNP and GA susceptibility is unknown. The present study is, to our knowledge, the first study to demonstrate an association between the common TLR4 gene rs2149356 polymorphism and GA susceptibility in a Chinese population with a case-control association analysis. We found that the frequencies of genotype and allele were significantly different between the GA and control groups, the frequencies of the TT genotype and T allele were significantly increased in patients with gout compared with control subjects. The TT genotype was associated with a significantly increased risk of GA. Adjusting multivariate logistic regression analysis for alcohol consumption, dietary factors, hypercholesterolemia, hyperuricemia, hypertension and BMI increased the significance of the association. Hence, the effect of rs2149356 on susceptibility to gout is independent of common risk factors. Moreover, no differences were observed between hyperuricemic and healthy subjects with respect to the frequencies of the genotypes or alleles. These data suggest that the T>G of rs2149356 SNP contributes to GA development. To confirm the association between innate immune genes SNPs and GA susceptibility, multicenter studies to replicate the findings in an independent population are needed.

Studies have demonstrated that the TLR4-IL1β signaling pathway is involved in the pathogenesis of gouty inflammation [12]. In our study, TLR4 mRNA level in PBMCs and IL1β level in serum were significantly increased in the GA group compared with the HC and HUA groups, which suggested that TLR4-IL1β signaling was activated in peripheral blood in GA patients. In AGA patients, PBMCs TLR4 mRNA and serum IL1β levels were much higher both in the TT homozygotes and GT heterozygotes than in the GG homozygotes, and they were higher in the TT homozygotes than in the GT heterozygotes. However, in NAGA patients, PBMCs TLR4 mRNA was significantly reduced both in the TT homozygotes and GT heterozygotes compared with the GG homozygotes. The present results suggest that, in acute gouty arthritis patients, the TT genotype of TLR4 gene rs2149356 is associated with higher PBMCs TLR4 mRNA and serum IL1β levels compared with the GG genotype, and that the TT genotype is associated with decreased TLR4 mRNA expression in non-acute gouty arthritis patients. These results indicate that TLR4 gene rs2149356 SNP might be involved in the regulation of TLR4 mRNA expression and IL1β levels in GA patients. The rs2149356 is located in intron 4 of the TLR4 gene. It is possible that the intronic sequence regulates the expression of the TLR4 gene and that rs2149356 polymorphism plays a critical role in the gene expression process. TLR4 mRNA expression in acute and non-acute GA patients shows an inverse associations. There might be a regulatory mechanism involving negative feedback in non-acute gout. Although we have found no reports that a polymorphism differentially regulates mRNA expression according to the disease condition, we can not exclude this possibility. More samples and functional tests are required to confirm our results. It is well worth notice that no differences in TLR4 mRNA or serum IL1β were observed in peripheral blood from the different genotypes in the healthy or hyperuricemic individuals. Thus, we speculate that rs2149356 SNP might impact TLR4 mRNA expression only when TLR4-IL1β signaling was activated; however, the concrete mechanism is unclear.

Based on the above results, we speculate that the TLR4 gene rs2149356 polymorphism might contribute to GA development by regulating the expression of TLR4-IL11β signaling pathway molecules. To confirm that the above findings are real, functional tests are considered for further study.

It should be noted that, significant differences in HDL, LDL, apoA1 and GLOB levels were observed among GA patients of the three genotypes, whereas no differences in these parameters were found in healthy or hyperuricemic subjects among the three genotypes. In our cohort study, HDL is significantly decreased but LDL increased in GA patients compared with both in hyperuricemic and healthy subjects. In GA patients, serum HDL and apoA1 were significantly increased, but serum LDL was significantly decreased, in patients with the TT genotype of the TLR4 gene rs2149356 compared with the GG genotype. These results indicate that TLR4 gene rs2149356 SNP might also be involved in the regulation of serum lipid metabolism, having the TT genotype might protect GA patients from developing abnormal lipid metabolism. GLOB includes in total immunoglobulins such as immunoglobulin G, A, M, which is produced by plasma cells after B lymphocytes activated. We found that serum GLOB was significantly increased in GA patients compared with both in hyperuricemic and healthy subjects. It is suggested that B lymphocytes are activated in the GA patients and that adaptive immunity might be involved in the pathogenesis of GA. GLOB level was much higher in patients with TT genotype than that in GG or GT genotypes, and no significant difference was observed between GG and GT genotypes in GA patients. These results indicate that the TLR4 gene rs2149356 SNP might also participate in the regulation of adaptive immunity in GA patients.

In conclusion, our findings indicate that the TLR4 gene rs2149356 SNP is associated with gout susceptibility, and might be involved in regulation of innate and adaptive immunity in gouty inflammation, that is might be participated in the regulating serum lipid metabolism in GA patients. More samples, as well as functional tests, are required to shed light on the association between innate immune genes SNPs and GA susceptibility; and to conclusively determine whether these SNPs regulate inflammation, immunity and/or lipid metabolism.
